# A Rev–CBP80–eIF4AI complex drives Gag synthesis from the HIV-1 unspliced mRNA

**DOI:** 10.1093/nar/gky851

**Published:** 2018-09-20

**Authors:** Daniela Toro-Ascuy, Bárbara Rojas-Araya, Francisco García-de-Gracia, Cecilia Rojas-Fuentes, Camila Pereira-Montecinos, Aracelly Gaete-Argel, Fernando Valiente-Echeverría, Théophile Ohlmann, Ricardo Soto-Rifo

**Affiliations:** 1 Laboratory of Molecular and Cellular Virology, Virology Program, Institute of Biomedical Sciences, Faculty of Medicine, Universidad de Chile, Santiago, Chile; 2 INSERM U1111, CIRI, Lyon F-69364, France; 3 Ecole Normale Supérieure de Lyon, Lyon F-69364, France

## Abstract

Gag synthesis from the full-length unspliced mRNA is critical for the production of the viral progeny during human immunodeficiency virus type-1 (HIV-1) replication. While most spliced mRNAs follow the canonical gene expression pathway in which the recruitment of the nuclear cap-binding complex (CBC) and the exon junction complex (EJC) largely stimulates the rates of nuclear export and translation, the unspliced mRNA relies on the viral protein Rev to reach the cytoplasm and recruit the host translational machinery. Here, we confirm that Rev ensures high levels of Gag synthesis by driving nuclear export and translation of the unspliced mRNA. These functions of Rev are supported by the CBC subunit CBP80, which binds Rev and the unspliced mRNA in the nucleus and the cytoplasm. We also demonstrate that Rev interacts with the DEAD-box RNA helicase eIF4AI, which translocates to the nucleus and cooperates with the viral protein to promote Gag synthesis. Finally, we show that the Rev/RRE axis is important for the assembly of a CBP80-eIF4AI complex onto the unspliced mRNA. Together, our results provide further evidence towards the understanding of the molecular mechanisms by which Rev drives Gag synthesis from the unspliced mRNA during HIV-1 replication.

## INTRODUCTION

Human Immunodeficiency Virus type-1 (HIV-1) gene expression is a complex process that leads to the synthesis of fifteen proteins from one single primary transcript (1,2). Once the proviral DNA has been integrated into the host cell genome, the RNA polymerase II drives the synthesis of a 9-kb, capped and polyadenylated pre-mRNA that undergoes alternative splicing generating more than 100 different transcripts classified into three main populations (3,4). The so-called 2-kb multiply spliced transcripts code for the key regulatory proteins Tat and Rev and the accessory protein Nef and are the dominant viral mRNA species at early stages of viral gene expression (1,2,5). Unlike cellular mRNAs or the 2-kb transcripts, which are spliced to completion before they exit the nucleus, HIV-1 and other complex retroviruses produce an important fraction of viral transcripts that remain incompletely spliced (2,6). These 4-kb transcripts are expressed during the intermediate phase of gene expression and are used for the synthesis of the envelope glycoprotein (Env) and the accessory proteins Vif, Vpr and Vpu (2,6). Finally, the full-length 9-kb pre-mRNA in its unspliced form also reaches the cytoplasm to be used as an mRNA template during the late stages of viral gene expression for the synthesis of the major structural proteins Gag and Gag-Pol (1,2,6).

Gene expression in eukaryotic cells occurs through the intricate connection of different processes including transcription, splicing, nuclear export, translation and mRNA decay and is regulated by the specific recruitment of nuclear proteins that together form the messenger ribonucleoprotein (mRNP) complex (7–9). As such, the early binding of the nuclear cap-binding complex (CBC) to the 5′-end cap structure and the splicing-dependent recruitment of the exon-junction complex (EJC) onto the mRNA have been shown to increase the rates of nuclear export and translation of spliced transcripts (10–20). Eukaryotic cells have also evolved quality control mechanisms ensuring that only properly processed mRNAs reach the cytoplasm and are decoded by the translational machinery. These mechanisms include the EJC-dependent degradation of transcripts containing premature termination codons through nonsense-mediated decay (NMD) or the NXF1-dependent nuclear retention of unspliced transcripts mediated by the nucleoporin Tpr (21–25). Consistent with these cellular quality control mechanisms, it has been widely reported that viral intron-containing transcripts including the 4-kb and the 9-kb mRNA produced during HIV-1 replication are retained and degraded in the host cell nucleus unless the viral protein Rev is present (26–30). As such, Rev has been proposed to promote HIV-1 gene expression from its target transcripts by (i) avoiding mRNA degradation (26,31); (ii) promoting nuclear export (31–33) or by (iii) promoting translation (34,35). In this study, we developed Rev mutant proviruses and confirmed that Rev is required for both nuclear export and translation of the HIV-1 unspliced mRNA. We show that the nuclear cap-binding complex subunit CBP80 and the translation initiation factor eIF4AI associate with Rev and the unspliced mRNA to promote Gag synthesis. We also demonstrate that the Rev/RRE axis is important for the assembly of a CBP80-eIF4AI complex onto the unspliced mRNA. Together, our work provides further insights into the molecular mechanism by which Rev drives Gag synthesis from the HIV-1 unspliced mRNA.

## MATERIALS AND METHODS

### DNA constructs

The pNL4.3 and pNL4.3R proviruses were previously described (36,37). These vectors were digested with NheI and subjected to a 20 min polymerization reaction at 72°C using the Phusion^®^ High-Fidelity DNA polymerase (New England Biolabs) in order to create a frameshift that generates a premature stop codon within the *env* gene. The resulting vectors were ligated with the T4 DNA ligase and transformed into *Escherichia coli* DH5α. To create the pNL4.3-ΔRev and pNL4.3R-ΔRev vectors, the above vectors were digested with BamHI and subjected to the same polymerization/ligation reaction to create a frameshift within the *rev* gene previously shown to abolish expression of a functional protein (28). The pCMV-NL4.3R and pCMV-NL4.3R-ΔRev vectors were obtained by replacing the FspAI/BssHII fragment of the corresponding vector by the CMV IE promoter amplified from the pCIneo vector (Promega) as we previously reported (38). The pNL4.3-CTE and pNL4.3R-CTE were previously described (38,39). The pCDNA-Flag-Rev vector was previously described (22). pCDNA-d2EGFP vector was generated by inserting the d2EGFP ORF into pCDNA3.1 (Life Technologies). pCDNA HIV-1 5'-UTR and pCDNA β-globin 5'-UTR were previously described (40). The dl HIV-1 IRES vector was previously described (41). The pCIneo-HA-eIF4GI, -eIF4A, -eIF4E and -eIF3g were previously described (42). The pCMV-myc-eIF4E and -CBP80 were previously described (43). The pBSK-Gag/Pol used for the generation of biotinylated probes was previously described (44).

### Cell culture and DNA transfection

HeLa cells and human microglia (C20 cells) (45) were maintained in DMEM (Life Technologies) supplemented with 10% FBS (Hyclone) and antibiotics (Hyclone) at 37°C and a 5% CO_2_ atmosphere. H9 T-lymphocytes (46) and THP-1_ATCC_ monocytes (47) were maintained in RPMI 1640 (Life Technologies) supplemented with 10% FBS (Hyclone) and antibiotics (Hyclone) at 37°C and a 5% CO_2_ atmosphere. Cells were transfected using linear PEI ∼25 000 Da (Polysciences) prepared as described previously (48). Cells were transfected using a ratio μg DNA/μl PEI of 1/15.

### Analysis of Renilla and firefly luciferase activities

Renilla luciferase activity was determined using the Renilla Reporter Assay System (Promega) and Renilla/firefly luciferase activities were determined using the Dual-Luciferase^®^ Reporter Assay System (Promega) in a GloMax^®^ 96 microplate luminometer (Promega).

### Western blot

Cells extracts from transfected cells were prepared by lysis with RIPA buffer and 20 μg of total protein were subjected to 10% SDS-PAGE and transferred to an Amersham Hybond™-P membrane (GE Healthcare). Membranes were incubated with an HIV-1 p24 monoclonal antibody diluted to 1/1000 (49), a rabbit anti-Flag antibody (Sigma-Aldrich) diluted to 1/1000 or and HRP-conjugated anti-actin antibody (Santa Cruz Biotechnologies) diluted to 1/750. Upon incubation with the corresponding HRP-conjugated secondary antibody (Santa Cruz Biotechnologies) diluted to 1/1000, membranes were revealed with the ECL substrate (Cyanagene) using a C-Digit digital scanner (Li-Cor).

### RNA extraction and RT-qPCR

Cytoplasmic RNA extraction and RT-qPCR from cytoplasmic RNA were performed essentially as recently described (44). Briefly, cells were washed intensively with PBS, recovered with PBS-EDTA 10 mM and lysed for 1–2 min at room temperature with 200 μl of buffer [10 mM Tris–HCl pH 8.0, 10 mM NaCl, 3 mM MgCl_2_, 1 mM DTT, 0.5% NP40 and 2 mM of vanadyl-ribonucleoside complex (VRC) (New England Biolabs)]. Cell lysates were centrifuged at 5000 rpm for 5 min at 4°C and supernatant containing the cytoplasmic fraction were recovered and RNA extraction was carried out by adding 1 ml of TRIzol™ (Thermo Fisher) as indicated by the manufacturer. Cytoplasmic RNAs (1 μg) were reverse-transcribed using the High Capacity RNA-to-cDNA Master Mix (Life Technologies). For quantitative PCR, a 20-μl reaction mix was prepared with 5 μl of template cDNAs (previously diluted to 1/10), 10 μl of FastStart Universal SYBR Green Master (Rox) (Roche), 0.2 μM of sense and antisense primers and subjected to amplification using the Rotorgen fluorescence thermocycler (Qiagen). The GAPDH housekeeping gene was amplified in parallel to serve as a control reference. Relative copy numbers of HIV-1 unspliced mRNA were compared to GAPDH or 18S rRNA using *x*^−ΔCt^ (where *x* correspond to the experimentally calculated amplification efficiency of each primer couple).

### Fluorescent in situ hybridization, immunofluorescence and confocal microscopy

Specific probes against the HIV-1 unspliced mRNA were generated by *in vitro* transcription using the pBSK-Gag/Pol vector and digoxin-11-UTP (Roche) as we previously described (44). The generated 5-kb transcript complementary to the Gag/Pol region of the unspliced mRNA was fragmented using the RNA fragmentation buffer (Thermo Scientific) in order to obtain probes of 100–200 nt in length following the supplier′s instructions. Fragmented probes were purified using the Agencourt AMPure XP magnetic beads (Beckman Coulter). RNA FISH was carried out as we recently described (44). Briefly, HeLa cells were cultured in a 12-well plate with coverslips and maintained and transfected with 1 μg of pNL4.3 or 1 μg of the corresponding expression vectors as indicated above. At 24 hpt, cells were washed twice with 1× PBS and fixed for 10 min at room temperature with 4% paraformaldehyde. Cells were subsequently permeabilized for 10 min at room temperature with 0.2% Triton X-100 and hybridized overnight at 37°C in 200 μl of hybridization mix (10% dextran sulfate, 2 mM VRC, 0.02% RNase-free BSA, 50% formamide, 300 μg tRNA and 120 ng of 11-digoxigenin-UTP probes) in a humid chamber. Cells were washed with 0.2× SSC/50% formamide during 30 min at 50°C and then incubated three times with antibody dilution buffer (2X SSC, 8% formamide, 2 mM vanadyl-ribonucleoside complex, 0.02% RNase-free BSA). Mouse anti-digoxin and rabbit anti-HA (Sigma Aldrich) primary antibodies diluted to 1/100 in antibody dilution buffer were added for 2 h at room temperature. After three washes with antibody dilution buffer, cells were incubated for 90 min at room temperature with anti-mouse Alexa 488 and anti-rabbit Alexa 594 antibodies (Molecular Probes) diluted at 1/500. Cells were washed three times in wash buffer (2× SSC, 8% formamide, 2 mM vanadyl-ribonucleoside complex), twice with 1× PBS, incubated with DAPI (0.3 μg/ml in PBS) (Life Technologies) for 1 min at room temperature, washed three times with 1× PBS, three times with water and mounted with Fluoromount (Life Technologies). Images were obtained with a TCS SP8 Confocal Microscope (Leica Microsystems) or a Zeiss LSM 800 Confocal Microscope (Zeiss) and processed using FIJI/ImageJ (NIH).

### Proximity ligation assay (PLA)

PLA (50), was carried out using the DUOLINK II In Situ kit (Sigma-Aldrich) and PLA probe anti-mouse minus and PLA probe anti-rabbit plus (Sigma-Aldrich) as we have previously described (38,51). Briefly, PFA-fixed HeLa cells were pre-incubated with blocking agent for 30 min at room temperature. Primary antibodies were added at a dilution of 1/100 (mouse anti-HA, Santa Cruz Biotechnologies) and 1/200 (rabbit anti-Flag, Sigma-Aldrich) in 40 μl DUOLINK antibody diluent and incubated at 37°C for 1 h. Samples were washed three times with PBS for 5 min each and secondary antibodies (DUOLINK anti-rabbit PLA-plus probe and DUOLINK anti-mouse PLA-minus probe) were added and incubated at 37°C for 1 h. Ligation and amplification reactions were performed following the same protocol described in (51). Samples were incubated with DAPI (0.3 μg/ml in PBS) (Life Technologies) for 1 min at room temperature, washed three times with PBS, three times with water and mounted with Fluoromount (Sigma Aldrich). Images were obtained with a TCS SP8 Confocal Microscope (Leica Microsystems) or a Zeiss LSM 800 Confocal Microscope (Zeiss) and processed using FIJI/ImageJ (NIH).

### In situ hybridization coupled to PLA (ISH-PLA)

The ISH-PLA protocol was developed by mixing the RNA-FISH and PLA protocols described above. Briefly, PFA-fixed HeLa cells growing on coverslips were permeabilized for 10 min at RT with 0.2% Triton X-100 and hybridized overnight at 37°C in 200 μl of hybridization mix (10% dextran sulphate, 2 mM vanadyl–ribonucleoside complex, 0.02% RNase-free bovine serum albumin, 50% formamide, 300 mg of tRNA and 120 ng of 11-digoxigenin-UTP probes) in a humid chamber. Cells were washed with 0.2× SSC/50% formamide during 30 min at 50°C and incubated with blocking agent for 30 min to room temperature. Cells were then incubated three times with antibody dilution buffer (2× SSC, 8% formamide, 2 mM vanadyl–ribonucleoside complex and 0.02% RNase-free bovine serum albumin). Mouse anti-digoxin and rabbit anti-protein of interest primary antibodies diluted to 1/100 in antibody dilution buffer were added for 2 h at room temperature. After three washes with antibody dilution buffer and two washes with PBS for 5 min each, the secondary antibodies (DUOLINK anti-rabbit PLA-plus probe, DUOLINK anti-mouse PLA-minus probe) were added and incubated at 37°C for 1 h. The ligation and amplification reactions were performed following the same protocol described above. Thereafter, coverslips were incubated with a solution of DAPI (0.3 μg/ml in PBS) (Life Technologies) for 1 min at room temperature, washed three times with PBS, three times with water and mounted with Fluoromount (Sigma Aldrich). Images were obtained with a TCS SP8 Confocal Microscope (Leica Microsystems) or a Zeiss LSM 800 Confocal Microscope (Zeiss) and processed using FIJI/ImageJ (NIH).

## RESULTS

### Rev promotes nuclear export and translation of the unspliced mRNA

**Figure 1.**
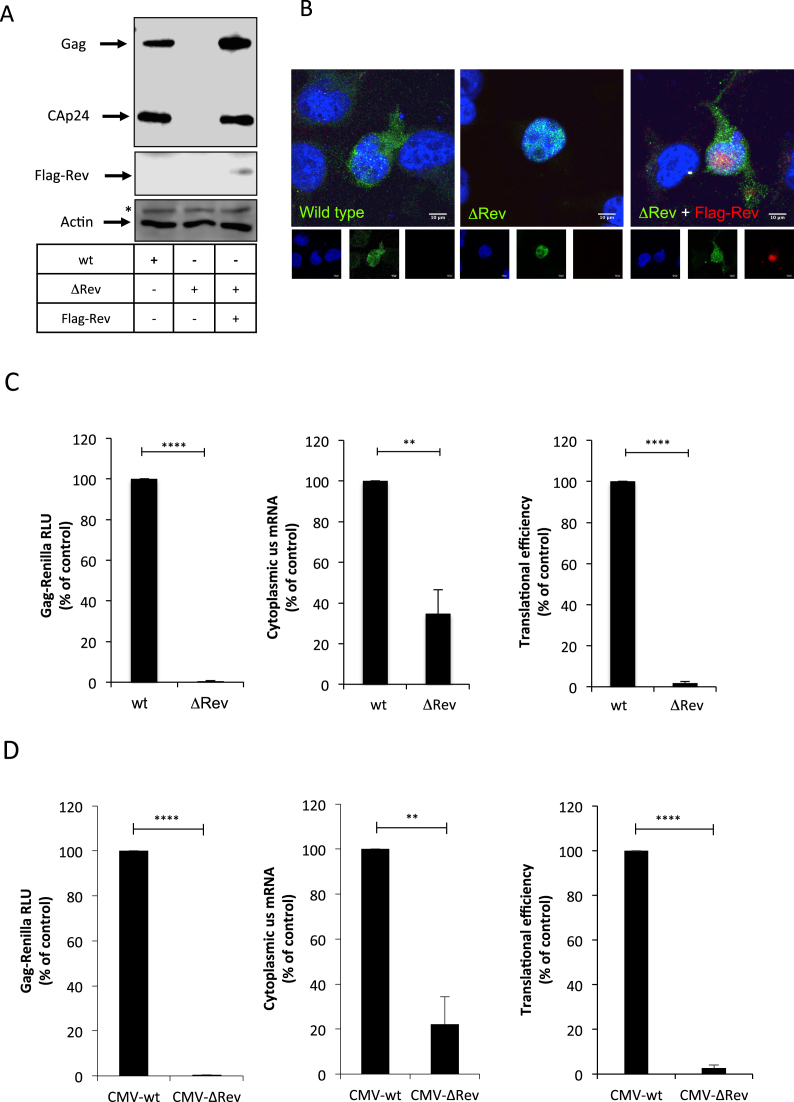
HIV-1 Rev promotes nuclear export and translation of the unspliced mRNA. (**A**) HeLa cells were transfected with 0.3 μg of pNL4.3-wt (wt), pNL4.3-ΔRev (ΔRev) or pNL4.3-ΔRev together with 0.1 μg of the pCDNA-Flag-Rev vector as described in materials and methods (pCDNA-d2EGFP was used as a control when pCDNA-Flag-Rev was not included). At 24 hpt, cell extracts were used to detect Gag and Flag-Rev by Western blot. Actin was used as a loading control. * Denotes an unspecific band detected with the anti-actin-HRP antibody. (**B**) HeLa cells were transfected as above and were subjected to RNA FISH and laser scan confocal microscopy analyzes as described in materials and methods. The unspliced mRNA is shown in green and Flag-Rev in red. Scale bar 10 μm. (**C**) HeLa cells were transfected with 0.3 μg of pNL4.3R (wt) or pNL4.3R-ΔRev (ΔRev) proviruses as described in materials and methods. At 24 hpt, cell extracts were prepared for Gag-Renilla luciferase activity measurement and for cytoplasmic RNA extraction and RT-qPCR analyzes. Results for Gag synthesis (left panel), cytoplasmic unspliced mRNA (middle panel) and translational efficiency (right panel) were normalized to the wild type provirus (arbitrary set to 100%) and presented as the mean ± SD of three independent experiments (**P < 0.01; ****P < 0.0001, *t*-test). (**D**) HeLa cells were transfected with 0.3 μg of pCMV-NL4.3R-wt (CMV-wt) or pCMV-NL4.3R-ΔRev (CMV-ΔRev) proviruses as described in materials and methods. At 24 hpt, cell extracts were prepared for Gag-Renilla activity measurement and for cytoplasmic RNA extraction and RT-qPCR analyzes. Results for Gag synthesis (left panel), cytoplasmic unspliced mRNA (middle panel) and translational efficiency (right panel) were normalized to the wild type provirus (arbitrary set to 100%) and presented as the mean +/- SD of three independent experiments (**P < 0.01; ****P < 0.0001, *t*-test).

The viral protein Rev promotes gene expression from the unspliced transcript by acting at the post-transcriptional level but the precise mechanism by which this occurs remains unclear (52–54). This prompted us to conduct a study aimed to gain further insights into the function of Rev on HIV-1 gene expression during viral replication. Thus, we used the pNL4.3 proviral DNA to introduce a frameshift within the *rev* gene previously shown to abolish the expression of a functional protein (28). Consistent with a critical role of Rev in the post-transcriptional regulation of the unspliced mRNA, we observed that Gag synthesis was abolished in the absence of Rev and restored upon Rev expression *in trans* (Figure 1A). In agreement with several previous reports (31–33), we observed that most of the unspliced mRNA is retained in the nucleus in the absence of Rev (Figure 1B, compare wild type and ΔRev). The cytoplasmic signal of the unspliced mRNA was recovered when Rev was expressed *in trans* (Figure 1B, see ΔRev + Flag-Rev), which is consistent with an important role of Rev in nuclear export. However, it has been reported that Rev is also important for translation of the unspliced mRNA during viral replication (34,35). Thus, in order to quantify the impact of Rev on gene expression from the unspliced mRNA we used the pNL4.3R reporter provirus to generate a ΔRev version as described above (see materials and methods). Transfection of pNL4.3R-ΔRev in HeLa cells, T-cells (H9 cells), monocytes (THP-1 cells) or human microglia (C20 cells) resulted in very low levels of Gag-Renilla expression when compared to the wild type provirus indicating that our reporter proviruses can be used to quantify the effects of Rev on gene expression from the unspliced mRNA (Supplementary Figure S1A). Transfection of the pNL4.3R-ΔRev together with a Flag-Rev expressing vector restored Gag synthesis to the wild type levels indicating that the defects in Gag-Renilla expression observed were exclusively due to the absence of Rev (Supplementary Figure S1B). Thus, we used the pNL4.3R-wt and -ΔRev proviruses to quantify the effects of Rev on Gag synthesis, cytoplasmic levels of the unspliced mRNA and its translational efficiency in HeLa cells as we have previously reported (37,38,42,44,55,56). As observed above, Gag production was almost abolished in the absence of Rev (Figure 1C, left panel). Consistent with its previously described role in nuclear export of the unspliced transcript (26,31–33), we observed that the cytoplasmic levels of the unspliced mRNA were reduced by 3-fold in the absence of Rev (Figure 1C, middle panel). Although these results differ from those presented in Figure 1B in which no genomic RNA could be detected in the cytoplasm, it has been proposed that an important fraction of the unspliced transcript reach the cytoplasm in the absence of Rev but remains trapped into a ribonucleoprotein complex inaccessible to the probes used during *in situ* hybridization (31). It should be mentioned that our cytoplasmic fractions were devoid of pre-GADPH mRNA discarding any contamination with nuclear RNA (Supplementary Figure S1C). However, this important decrease in the cytoplasmic levels of the unspliced transcript does not account for the dramatic reduction in Gag synthesis (>100-fold) indicating that most of the unspliced mRNAs that reach the cytoplasm in the absence of Rev are not translated (Figure 1C, right panel). Together, these data confirm that the function of Rev during viral replication is not restricted to nuclear export since it is also critical for translation.

While the role of Rev in nuclear export has been largely characterized (52), the molecular mechanism by which Rev promotes translation of the unspliced transcript is not very well understood (57). In this sense, it was previously reported that Rev is able to promote translation of a reporter RNA by an unknown mechanism involving the binding to an RNA motif (A-loop) present within SL1 of the unspliced mRNA 5′-UTR (53,58) (Supplementary Figure S1D). Consistent with this previous work, we observed that expression of a Renilla luciferase-based monocistronic vector harboring the 5′-UTR of the unspliced transcript (but not that of the human β-globin) was stimulated up to 2-fold in the presence of Rev (Supplementary Figure S1E). Such a stimulation was not observed when IRES-driven translation was analyzed with a previously described bicistronic vector (41), suggesting that the effect of Rev on the 5′-UTR is exerted at the level of cap-dependent translation (Supplementary Figure S1F).

Although the 2-fold stimulation observed with the reporter construct is consistent with the previous report (53), it does not account for the strong dependence of Rev for unspliced mRNA translation observed in the context of a full-length provirus (Figure 1A and C), suggesting that the 5′-UTR is not the only molecular determinant involved in Rev-mediated translation of the unspliced mRNA. In order to confirm this hypothesis, we constructed a ΔRev version of the CMV-pNL4.3R vector, a reporter provirus lacking the 5′-LTR and most of the 5′-UTR but containing the CMV IE promoter (38). This proviral DNA is expected to produce an unspliced mRNA that only contains the last 79 nucleotides of the wild type 5′-UTR, therefore lacking the Rev binding site previously described in the A-loop of SL1. We transfected the CMV-pNL4.3R and CMV-pNL4.3R-ΔRev vectors and analyzed the role of Rev in Gag synthesis, cytoplasmic levels of the unspliced mRNA and translation as described above. Interestingly, we observed a strong dependence for Rev in translational efficiency of the unspliced transcript regardless of whether the entire 5′-UTR was driving ribosome recruitment or not (Figure 1D). Together, these data suggest that the previously described Rev-binding site present within the 5′-UTR is not the major molecular determinant involved in the translational stimulation mediated by Rev in the context of viral replication. Given the fact that our CMV-pNL4.3R provirus also lacks major determinants required for IRES-driven translation (41), these data also suggest that Rev promotes cap-dependent translation.

### The CBC subunit CBP80 interacts with Rev and promotes nuclear export and translation of the unspliced mRNA

**Figure 2.**
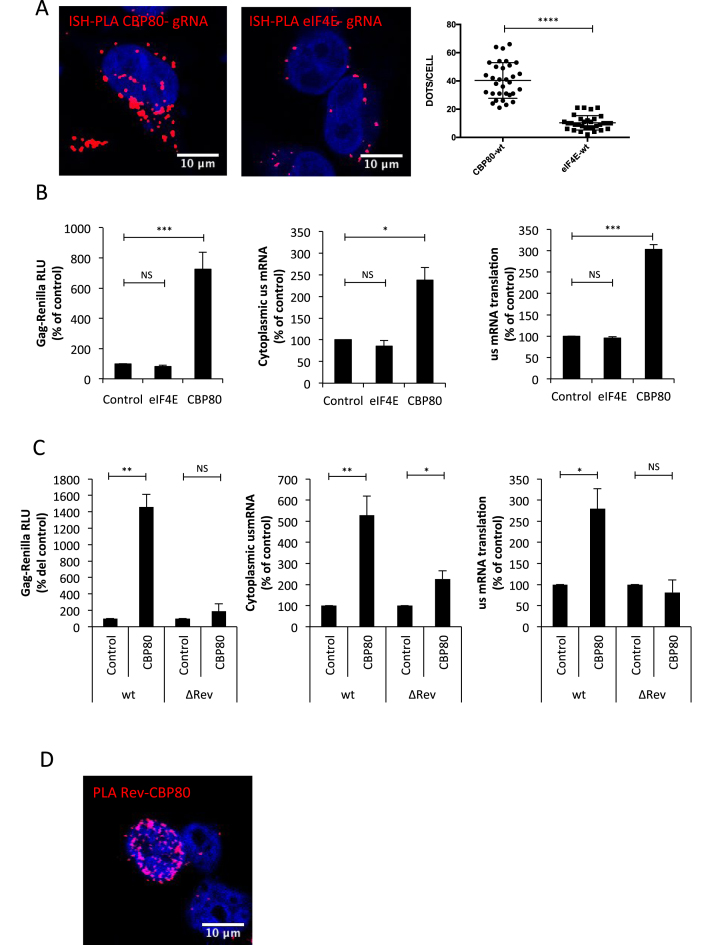
CBP80 cooperates with the functions of Rev on Gag synthesis from the unspliced mRNA. (**A**) HeLa cells were transfected with 1 μg pNL4.3-wt together with 1 μg pCMV-myc-CBP80 or 1 μg pCMV-myc-eIF4E. At 24 hpt, the interaction between the unspliced mRNA and the myc- tagged protein was analyzed by the ISH-PLA protocol described in materials and methods. Red dots indicate the interactions between the unspliced mRNA and the corresponding myc-tagged protein. Scale bar 10 μm. A quantification of the dots/cell in each condition is presented on the right (****P < 0.0001, Mann–Whitney test). (**B**) HeLa cells were transfected with 0.3 μg of pNL4.3R or pNL4.3R ΔRev proviruses together with 1 μg of pCMV-myc-CBP80 or pCMV-myc-eIF4E (pCMV-myc-d2EGFP was used as control). At 24 hpt, cell extracts were prepared for Gag-Renilla activity measurement and for cytoplasmic RNA extraction and RT-qPCR analyzes. Results for Gag synthesis (left panel), cytoplasmic unspliced mRNA (middle panel) and translational efficiency (right panel) were normalized to the wild type provirus (arbitrary set to 100%) and presented as the mean ± SD of three independent experiments (*P < 0.05; ***P < 0.001 and NS; non-significant, *t*-test). (**C**) HeLa cells were transfected with 0.3 μg of pNL4.3R or pNL4.3R ΔRev proviruses together with 1 μg of pCMV-myc-CBP80 (pCMV-myc-d2EGFP was used as control) as described in materials and methods. At 24 hpt, cell extracts were prepared for Gag-Renilla activity measurement and for cytoplasmic RNA extraction and RT-qPCR analyzes. Results for Gag synthesis (left panel), cytoplasmic unspliced mRNA (middle panel) and translational efficiency (right panel) were normalized to the wild type provirus (arbitrary set to 100%) and presented as the mean ± SD of three independent experiments. (*P < 0.05; **P < 0.01 and *NS;* non-significant, *t*-test). (**D**) HeLa cells were transfected with 1 μg pCDNA-Flag-Rev and 1 μg pCDNA-V5-CBP80. At 24 hpt, the interaction between Flag-Rev and V5-CBP80 was analyzed by PLA. Red dots indicate interactions between both proteins. Scale bar 10 μm.

From data presented above, it seems that Rev promotes cap-dependent translation of the unspliced mRNA. In this regard, a previous report suggested that cap-dependent CBC-driven translation could ensure Gag synthesis during an HIV-1-induced inhibition of eIF4E activity (59). In agreement with this idea, we have previously shown that the cytoplasmic cap-binding protein eIF4E is excluded from a translation initiation mRNP containing the HIV-1 unspliced mRNA together with the RNA helicase DDX3 and translation initiation factors eIF4GI and PABPC1 (44). Thus, in order to determine whether the unspliced mRNA is associated to the CBC or eIF4E, we established a protocol based on *in situ* hybridization of digoxin-labeled probes directed to the unspliced mRNA coupled to the proximity ligation assay (ISH-PLA) in order to determine and quantify unspliced mRNA-protein interactions (Supplementary Figure S2A and materials and methods). This strategy has been successfully used to detect RNA:protein interactions in intact cells (60). By using our ISH-PLA protocol, we observed that the unspliced mRNA preferentially associates with the CBC subunit CBP80 rather than eIF4E (Figure 2A). Despite the fact that most of the CBP80 signal was present in the nucleus in RNA FISH-IF experiments performed in parallel (Supplementary Figure S2B), the interaction between the unspliced mRNA and the CBC subunit occurs predominantly in the cytoplasm suggesting that the unspliced mRNA remains associated to the CBC upon nuclear export. Signal intensity quantifications from the RNA FISH experiments performed in parallel revealed no differences in myc-tagged protein expression (Supplementary Figure S2C). To further investigate the role of CBP80 and eIF4E on gene expression from the unspliced mRNA, we independently overexpressed both proteins in HeLa cells and analyzed Gag synthesis, cytoplasmic unspliced mRNA levels and translational efficiency as described above. Consistent with a preferential association of the unspliced mRNA with CBP80, we observed that overexpression of the CBC subunit but not eIF4E results in a marked increase in Gag synthesis, which was due to an increase in both cytoplasmic accumulation and translation of the unspliced mRNA (Figure 2B). We observed that CBP80 overexpression only resulted in marginal (2-fold) stimulation of the unspliced mRNA in the cytoplasm when the ΔRev provirus was used suggesting that CBP80 cooperates with Rev during the post-transcriptional control of the unspliced mRNA (Figure 2C). Of note, Gag-Renilla activity from the wild type provirus was largely higher than that observed from the ΔRev provirus consistent with data presented in Figure 1 (data not shown). We also analyzed the effect of CBP80 overexpression on Vif (Rev-dependent) and Nef (Rev-independent) synthesis but we were not able to observe the same effects observed with Gag (Supplementary Figure S2D and E).

Since it was previously shown that CBP80 interacts with Rev *in vitro* (61), we wanted to evaluate whether this interaction also occurs in cells. Thus, we performed PLA and observed that Flag-tagged Rev interacts with both endogenous and V5-tagged CBP80 (Supplementary Figure S2F and Figure 2D).

Together, these results suggest that the unspliced mRNA is preferentially associated to the CBC and the CBC subunit CBP80 interacts and cooperates with Rev to promote nuclear export and translation of this viral transcript.

### DEAD-box helicase eIF4AI interacts with Rev and promotes Gag synthesis from the unspliced mRNA

**Figure 3.**
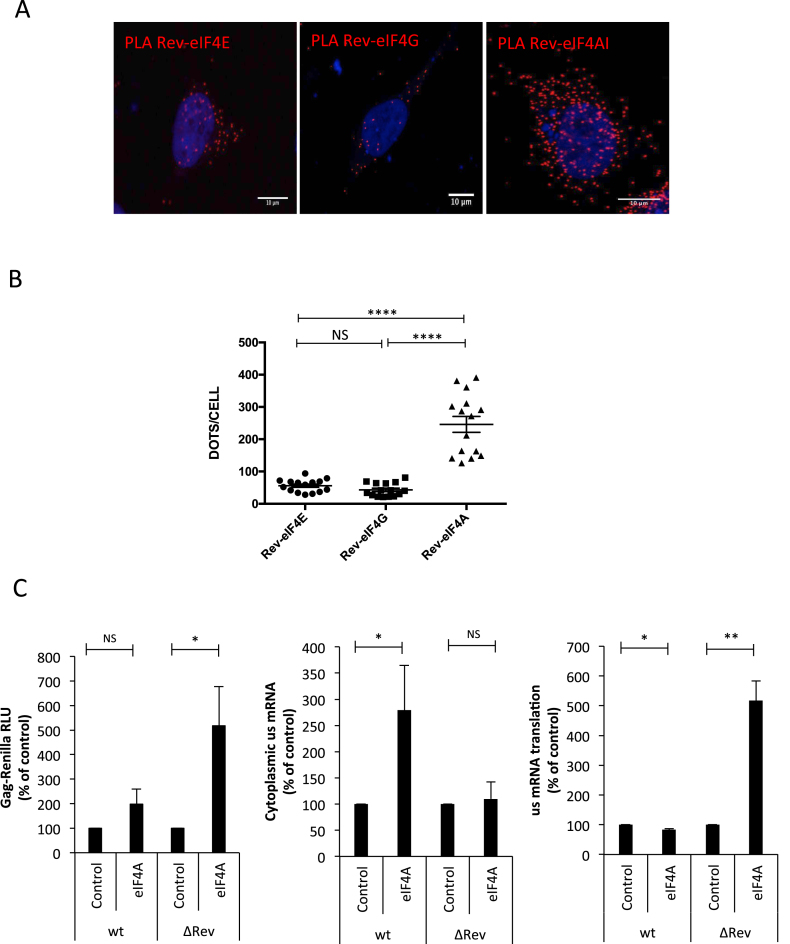
DEAD-box RNA helicase eIF4AI interacts with Rev and promotes Gag synthesis from the unspliced mRNA. (**A**) HeLa cells were transfected with 1 μg pCDNA-Flag-Rev together with 1 μg pCIneo-HA-eIF4E, pCIneo-HA-eIF4G or pCIneo-HA-eIF4AI. At 24 hpt, the interaction between Flag- and HA- tagged eIFs was analyzed by PLA. Red dots indicate interactions between Flag-Rev and the corresponding HA-tagged protein. Scale bar 10 μm. (**B**) Dots/cell for Rev-4E, Rev-4G and Rev-4A were quantified using ImageJ (****P < 0.0001, Mann–Whitney test). (**C**) HeLa cells were transfected with 0.3 μg of pNL4.3R-wt or pNL4.3R-ΔRev together with 1 μg of the pCIneo-HA-eIF4AI vector as described in materials and methods (pCIneo-HA-d2EGFP was used as a control). At 24 hpt, cell extracts were prepared for Gag-Renilla activity measurement and for cytoplasmic RNA extraction and RT-qPCR analyzes. Results for Gag synthesis (left panel), cytoplasmic unspliced mRNA (middle panel) and translational efficiency (right panel) were normalized to the wild type provirus (arbitrary set to 100%) and presented as the mean ± SD of three independent experiments (*P < 0.05; **P < 0.01 and *NS;* non-significant, *t*-test).

Having determined that the unspliced mRNA is preferentially associated with CBP80 and that this CBC subunit interacts with Rev, we were interested in identify additional translation initiation factors interacting with Rev that could be involved in Gag synthesis. Although the CBP20/80-dependent translation initiation factor (CTIF) was shown to be important for CBC-dependent translation (62), we observed that CTIF is rather a potent inhibitor of Gag synthesis (García-de-Gracia *et al.*, manuscript in preparation). Thus, we reasoned that Rev and CBP80 form an mRNP different from the canonical CBC, which is important for unspliced mRNA nuclear export and translation. Having shown that eIF4E does not affect translation of the unspliced mRNA (Figure 2A), we looked whether additional components of eIF4F such as eIF4GI and eIF4AI were associated with Rev. Indeed, eIF4GI was shown to interact with CBP80 and thus, we supposed that it could be recruited to the Rev–CBP80 complex (63). Interestingly, our PLA using HA-tagged versions of eIF4E, 4GI and 4AI together with Flag-tagged Rev revealed that Rev forms nuclear and cytoplasmic complexes with eIF4AI and at a much lesser extent with eIF4E and eIF4GI (Figure 3A and B). It should be mentioned that IF experiments performed in parallel revealed no differences in the intensity signals amongst HA-tagged eIFs and Flag-Rev indicating that the increased number of interactions observed between eIF4AI and Rev were not due to differences in the ectopic expression levels of the proteins (Supplementary Figure S3A and B). From these data, it could be speculated that Rev recruits eIF4AI to the unspliced mRNA in order to promote translation. Thus, in order to evaluate the involvement of eIF4AI in Rev activity, we overexpressed the RNA helicase and analyzed its impact on Gag synthesis, cytoplasmic unspliced mRNA levels and translation using our wild type and ΔRev reporter proviruses (Figure 3C). As expected, Gag-Renilla activity from the wild type provirus was much higher than that observed from the ΔRev provirus (data not shown). Interestingly, we observed that eIF4AI overexpression results in a 2- and 5-fold increase in Gag synthesis from the wild type and ΔRev reporter proviruses, respectively (Figure 3C, left panel). Surprisingly, analysis of the cytoplasmic unspliced mRNA levels upon eIF4AI overexpression revealed a 2- to 3-fold increase for the wild type provirus with no effects on the ΔRev provirus (Figure 3C, middle panel). More strikingly, we observed that translation from the ΔRev provirus was stimulated up to 6-fold by eIF4AI overexpression with no effects on translation from the wild type provirus (Figure 3C, right panel). It should be mentioned that treatment of cells with hippuristanol resulted in the inhibition of unspliced mRNA translation indicating that this RNA helicase also participates in this process (Supplementary Figure S3C). These results suggest that the presence of Rev will determine the process (nuclear export or translation) by which ectopically expressed eIF4AI promotes Gag synthesis from the unspliced mRNA.

### Rev regulates the association of CBP80 and eIF4AI to the unspliced mRNA

**Figure 4.**
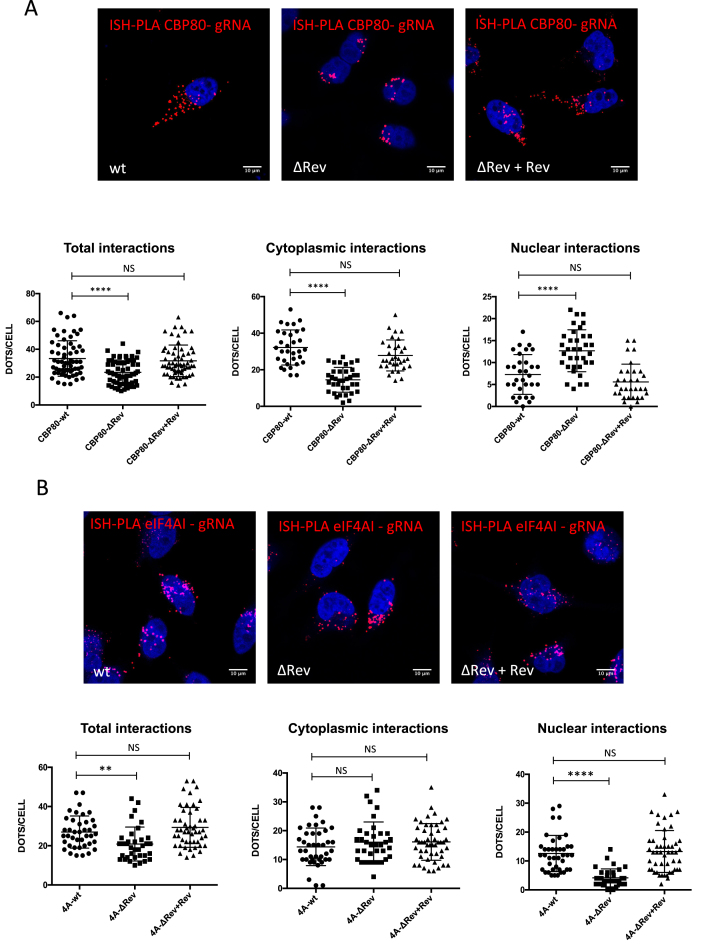
Rev promotes the recruitment of CBP80 and eIF4AI to the HIV-1 unspliced mRNA. (**A**) HeLa cells were transfected with 1 μg pNL4.3-wt, 1 μg pNL4.3 ΔRev or 1 μg pNL4.3 ΔRev + 0,3 μg pCDNA-Flag-Rev together with 1 μg pCMV-myc-CBP80. At 24 hpt, the interaction between unspliced mRNA and CBP80 was analyzed by ISH-PLA. Scale bar 10 μm (upper panel). Dots per cell quantifications for total unspliced mRNA-CBP80 interactions (left panel), cytoplasmic interactions (middle panel) and nuclear interactions (right panel) are presented below. All interactions were quantified using ImageJ (****P < 0.0001 and *NS*; non-significant, Mann–Whitney test). (**B**) HeLa cells were transfected with 1 μg of pNL4.3-wt, pNL4.3-ΔRev or 1 μg pNL4.3-ΔRev + 0,3 μg pCDNA-Flag-Rev together with 1 μg pCIneo-HA-eIF4AI. At 24 hpt, the interaction between the unspliced mRNA and eIF4AI was analyzed by ISH-PLA. Scale bar 10 μm (upper panel). Dots per cell for total unspliced mRNA–eIF4AI interactions (left panel), cytoplasmic interactions (middle panel) and nuclear interactions (right panel) are presented below. All interactions were quantified using ImageJ (**P < 0.01; ****P < 0.0001 and *NS*; non-significant, Mann–Whitney test) (lower panel).

Giving the fact that the presence of Rev modulates the activity of CBP80 and eIF4AI on Gag synthesis, we wanted to determine whether Rev was involved in the recruitment of these cellular proteins to the unspliced mRNA. For this, we quantified the interaction between the unspliced mRNA and CBP80 or eIF4AI in the presence and absence of Rev using our ISH-PLA protocol. Interestingly, we observed that the interactions between CBP80 and the unspliced mRNA were reduced in the ΔRev provirus but were restored upon expression of Rev *in trans* suggesting that Rev favors and/or stabilizes the association between CBP80 and the unspliced mRNA (Figure 4A, left panel). Analysis of the nuclear and cytoplasmic signals suggests that Rev is necessary to maintain the CBP80-unspliced mRNA interaction in the cytoplasm (Figure 4A, compare middle and right panels). Signal intensity analysis from RNA FISH experiments performed in parallel revealed no differences in CBP80 expression between each condition (Supplementary Figure S4A and B).

We also observed that the eIF4AI-unspliced mRNA interactions were reduced in the absence of Rev and restored when the viral protein was expressed *in trans* (Figure 4B, left panel). Interestingly, we noticed that while most of the interactions between the unspliced mRNA and eIF4AI in the cytoplasm are independent of Rev, the viral protein favors the interaction in the nucleus (Figure 4B, middle and right panels). Signal intensity analysis from RNA FISH experiments performed in parallel revealed no differences in eIF4AI expression between each condition (Supplementary Figure S4C and D). This observation is consistent with our data presented in Figure 3C in which ectopic expression of eIF4AI favors the accumulation of the unspliced mRNA in the cytoplasm in the presence of Rev and promotes translation in the ΔRev provirus. Indeed, we observed an increase in the nuclear signal of HA-eIF4AI with the wild type provirus that was absent with the ΔRev provirus suggesting that a fraction of the RNA helicase might translocate to the nucleus in the presence of Rev (Supplementary Figure S4C and E).

Together, these results suggest that Rev regulates the association of CBP80 and eIF4AI with the unspliced mRNA in the cytoplasm and the nucleus, respectively.

### The Rev/RRE axis favors the recruitment of a CBP80-eIF4AI complex onto the unspliced mRNA

**Figure 5.**
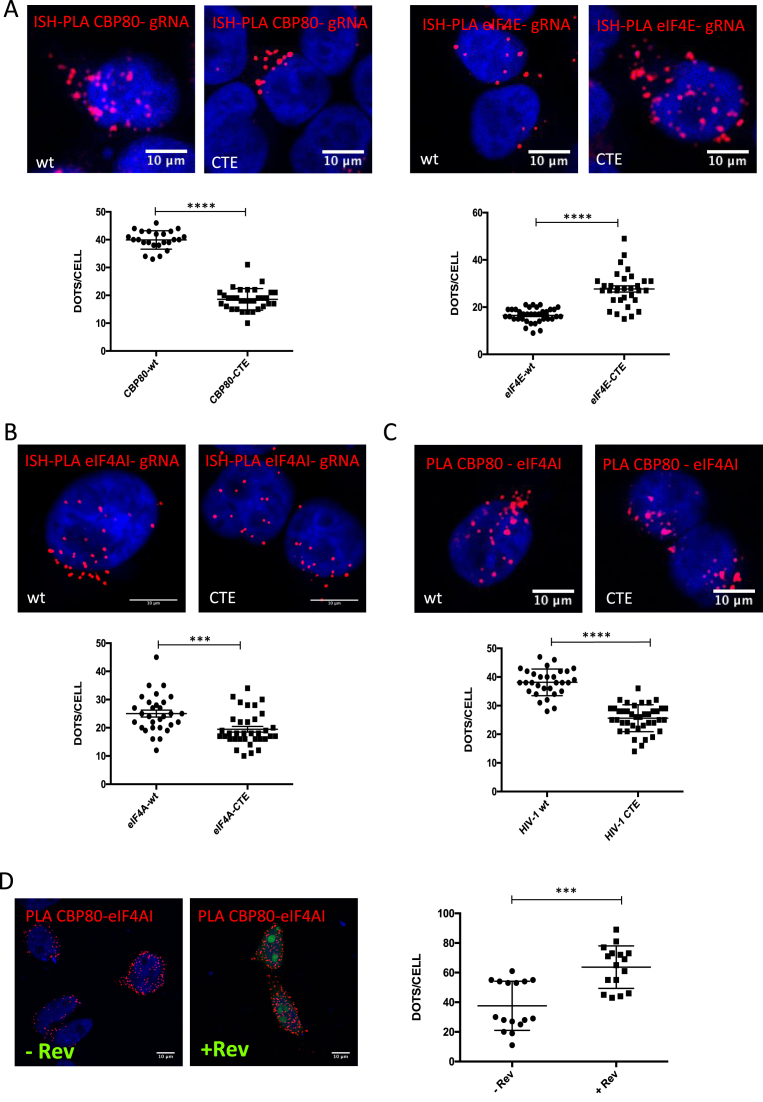
The Rev/RRE axis favors the recruitment of CBP80 and eIF4AI to the HIV-1 unspliced mRNA. (**A**) HeLa cells were transfected with 1 μg pNL4.3-wt or 1 μg pNL4.3-CTE together with 1 μg of pCMV-myc-CBP80 or pCMV-myc-eIF4E. At 24 hpt, the interaction between the unspliced mRNA and CBP80 or eIF4E was analyzed by ISH-PLA. Scale bar 10 mm (upper panels). Dots per cell quantifications for total unspliced mRNA-CBP80 or unspliced mRNA-eIF4E interactions are presented in the lower panels. Dots quantifications were performed with ImageJ (****P < 0.0001, Mann–Whitney test). (**B**) HeLa cells were transfected with 1 μg pNL4.3-wt or 1 μg pNL4.3-CTE together with 1 μg of pCIneo-HA-eIF4AI. At 24 hpt, the interaction between unspliced mRNA and eIF4AI was analyzed by ISH-PLA. Scale bar 10 mm (upper panel). Dots per cell quantifications for total unspliced mRNA-eIF4AI interactions are presented in the lower panel. Dots quantifications were performed with ImageJ (***P < 0.001 and *NS*; non-significant, Mann–Whitney test). (**C**) HeLa cells were transfected with 1 μg pNL4.3-wt or 1 μg pNL4.3-CTE together with 1 μg pCMV-myc-CBP80 and 1 μg pCIneo-HA-eIF4AI. At 24 hpt, the interaction between HA-eIF4AI and myc-CBP80 was analyzed by PLA (upper panels). Scale bar 10 μm. Dots per cell quantification of eIF4AI and CBP80 interactions were performed with ImageJ (****P < 0.0001, Mann–Whitney test). (**D**) HeLa cells were transfected with 1 μg pCMV-myc-CBP80 and 1 μg pCIneo-HA-eIF4AI together 1 μg pEGFP-Rev (pEGFP was used as a control). At 24 hpt, the interaction between HA-eIF4AI and myc-CBP80 was analyzed by PLA (left panels). Scale bar 10 μm. Dots per cell quantification of eIF4AI and CBP80 interactions were performed with ImageJ (***P < 0.001, Mann–Whitney test).

From our data presented above, it appears that Rev regulates Gag synthesis by associating with CBP80 and eIF4AI and regulating the association of these cellular proteins with the unspliced mRNA in the nucleus and the cytoplasm. Our data also suggest that Rev does not exert its functions at the 5′-UTR of the unspliced mRNA. Therefore, we sought to determine whether the Rev/RRE axis was important for the recruitment of these cellular proteins to the unspliced mRNA. For this, we took advantage of the pNL4.3-CTE provirus, which generates an unspliced mRNA that contains a mutated, non-functional, Rev/RRE axis but exits the nucleus through the Simian retrovirus-1 constitutive transport element (39,64). As expected, Rev expression *in trans* only promotes Gag synthesis of the ΔRev but not the CTE provirus (Supplementary Figure S5A). Consistent with data presented above, Rev expressed *in trans* promotes Gag synthesis from the ΔRev provirus by favoring nuclear export and translation of the unspliced mRNA (Supplementary Figure S5A). These data also confirm that Gag synthesis from the CTE provirus is completely independent of the presence of Rev. Thus, we first used the wild type and CTE proviruses in order to determine the involvement of the Rev/RRE axis in the association of the unspliced mRNA with components of the host translational machinery by ISH-PLA. We observed that while the unspliced mRNA from the wild type provirus preferentially associates with CBP80, it is preferentially bound to eIF4E when exported by the CTE (Figure 5A). We also observed that the presence of a functional Rev/RRE axis slightly favors the association of the unspliced mRNA with eIF4AI (Figure 5B) but not with eIF4GI or the eIF3 subunit eIF3g (Supplementary Figure S5B).

Thus, it appears that a functional Rev/RRE axis is important for the loading of a CBP80-eIF4AI complex onto the unspliced mRNA. However, although the interaction between CBP80 and eIF4AI was proposed some time ago (63), to our knowledge, it has not been experimentally demonstrated. Therefore, we analyzed the CBP80-eIF4AI interaction by PLA and determined whether the presence of a functional Rev/RRE axis was involved. Interestingly, we observed that both cellular proteins form a complex in cells and that the presence of the wild type provirus favors such an interaction (Figure 5C). Considering that our data indicates that Rev interacts with CBP80 and eIF4AI, we finally wanted to determine whether Rev was responsible of the increased interaction between both cellular proteins observed with the wild type provirus. For this, we performed PLA in the presence or absence of Rev and observed that indeed the viral protein favors the association between CBP80 and eIF4AI (Figure 5D).

Taken together, these data suggest that a functional Rev/RRE axis favors the assembly of a CBP80-eIF4AI complex as well as its loading onto the unspliced mRNA.

## DISCUSSION

Gag synthesis from the unspliced mRNA is a critical step during HIV-1 replication necessary for the efficient production of the viral progeny. Indeed, stoichiometric studies revealed that up to 5000 molecules of Gag are required to build one single viral particle (65), indicating that the unspliced mRNA needs to be efficiently expressed. However, since this viral mRNA contains functional introns, it must overcome surveillance mechanisms that induce its nuclear retention and degradation before it reaches the translational machinery in the cytoplasm to produce Gag (66). As such, understanding how the unspliced mRNA is efficiently exported from the nucleus and translated in the cytoplasm is not only critical to improve our knowledge on the molecular mechanisms driving viral gene expression but is also important to identify new pathways and/or interactions occurring within the cell or induced by the virus that could be targeted by novel antiretroviral drugs.

Although the unspliced mRNA associated to Rev, CRM1 and other co-factors does not resemble to a canonical mRNP that must be directed to the ribosomes upon nuclear export (i.e., an mRNA associated to cellular components such as TREX, NXF1 and the EJC), translation of the unspliced mRNA is highly efficient in cells and nuclear export across the Rev/CRM1 pathway is critical for ribosome recruitment (37,67). In this study, we provide evidence that the viral protein Rev acts as a nuclear imprint critical for nuclear export and translation of the unspliced mRNA (Figure 1). We reasoned that recruitment of Rev might serve as a platform for the spatiotemporal recruitment of host factors required to interconnect nuclear export and translation of the unspliced mRNA. In order to study the interaction between the unspliced mRNA and host proteins, we developed the ISH-PLA strategy, which allowed us to identify and quantify unspliced mRNA-protein interactions but also to determine the cellular location in which such interactions occur. In agreement with previous data (59), we observed that the HIV-1 unspliced mRNA exported through the Rev/RRE axis is preferentially associated to the CBC subunit CBP80 both in the nucleus and the cytoplasm (Figures 2 and 5). We also confirmed that CBP80 interacts with Rev and showed that the CBC subunit supports the activity of Rev in nuclear export and translation. Despite the fact that *in silico* data suggest that Rev should not interfere with the interaction between CBP80 and CBP20 (data not shown), it is still unknown whether CBP80 alone, or in the context of the CBC, is responsible of these functions. Moreover, our unpublished data also shows that CTIF, the CBP80/20-dependent translation initiation factor, is a potent inhibitor of Gag synthesis suggesting that CBP80 acts independently of a canonical CBC (Garcia-de-Gracia *et al.* manuscript in preparation). It was recently reported that the DEAD-box RNA helicase DDX3, which is critical for nuclear export and translation of the HIV-1 unspliced mRNA (38,42,44,68), promotes CBC-eIF3-mediated translation (69). These observations suggest that CBP80 (probably with Rev, eIF4A and DDX3) might promote Gag synthesis in the context of a non-canonical CBC that mediates the recruitment of the 40S ribosomal subunit. Interestingly, a recent study reported the existence of an alternative CBC formed by CBP80 and NCBP3, a novel cap-binding protein specifically associated to mRNA nuclear export (19). Thus, it would be of interest to determine whether the translating unspliced mRNA is indeed associated to the CBC and which of the cap-binding proteins, CBP20 or NCBP3, is bound to the cap structure of the viral transcript during translation. In this sense, the HIV-1 unspliced mRNA was shown to contain a m^2,2,7^GpppG trimethylated cap in a process catalyzed by the methyltransferase PIMT and dependent on the presence of Rev (70). It was also reported that increased cap trimethylation by PIMT overexpression was shown to promote Gag synthesis suggesting that a trimethylated cap favors polysome association of the unspliced mRNA (70). Since trimethylation reduces the affinity of CBP20 and eIF4E for the cap (71,72), it would be of interest to test the affinity of NCBP3 for m^2,2,7^GTP and whether this new cap-binding protein drives translation of the HIV-1 unspliced mRNA together with CBP80. It should be mentioned that our ISH-PLA analyses do not discard an association of the unspliced mRNA with the cap-binding protein eIF4E, which probably reflects the proportion of viral transcripts that contain monomethylated caps. Previous reports have shown that HIV-1 Gag synthesis and replication are maintained under inhibition of eIF4E-driven cap-dependent translation (59,73,74). Whether the Rev–CBP80–eIF4AI complex or the IRES-driven mechanism of ribosome recruitment are responsible of maintaining Gag synthesis under unfavorable conditions needs to be further investigated.

We also identified the DEAD-box RNA helicase eIF4AI as an additional partner of Rev (Figure 3) and observed that ectopic expression of eIF4AI promoted both the cytoplasmic accumulation of the unspliced mRNA or translation depending on whether Rev was present or not. Consistent with these observations, we observed that Rev promotes the interaction between eIF4AI and the unspliced mRNA in the nucleus (Figure 4). Since inhibition of eIF4AI/II function by hippuristanol treatment resulted in a strong inhibition of unspliced mRNA translation from the wild type provirus, we propose that the RNA helicase plays a dual role during viral replication by assisting Rev during nuclear export and by promoting unspliced mRNA translation independently of the viral protein. Further work is necessary to decipher the mechanism by which eIF4AI cooperates with Rev during nuclear export. We further showed that CBP80 associates with eIF4AI and this interaction was stimulated in the presence of the Rev/RRE axis (Figure 5). Despite to contain retained introns, retroviral unspliced mRNAs were shown to escape quality control mechanisms such as NMD (75). In this sense, it would be of interest to evaluate whether Rev and their retroviral counterparts are able to regulate the pioneer round of translation and NMD.

Last but not least, the small molecule ABX464, currently under a phase II clinical trial, was shown to interfere with the Rev–CBP80 interaction (76,77). Therefore, results presented here will be useful either for the better understanding of the mechanism of action of this small molecule or for the rational design of new drugs targeting the Rev–CBP80–eIF4AI complex.

## Supplementary Material

Supplementary Data
