# Supplementary material for: A Rev–CBP80–eIF4AI complex drives Gag synthesis from the HIV-1 unspliced mRNA
Source: Nucleic Acids Res. 2018 Sep 20;46(21):11539–52. doi: 10.1093/nar/gky851 (PMC6265489; doi:10.1093/nar/gky851)
Supplement: Supplementary Data [file gky851_supplemental_files.pdf]

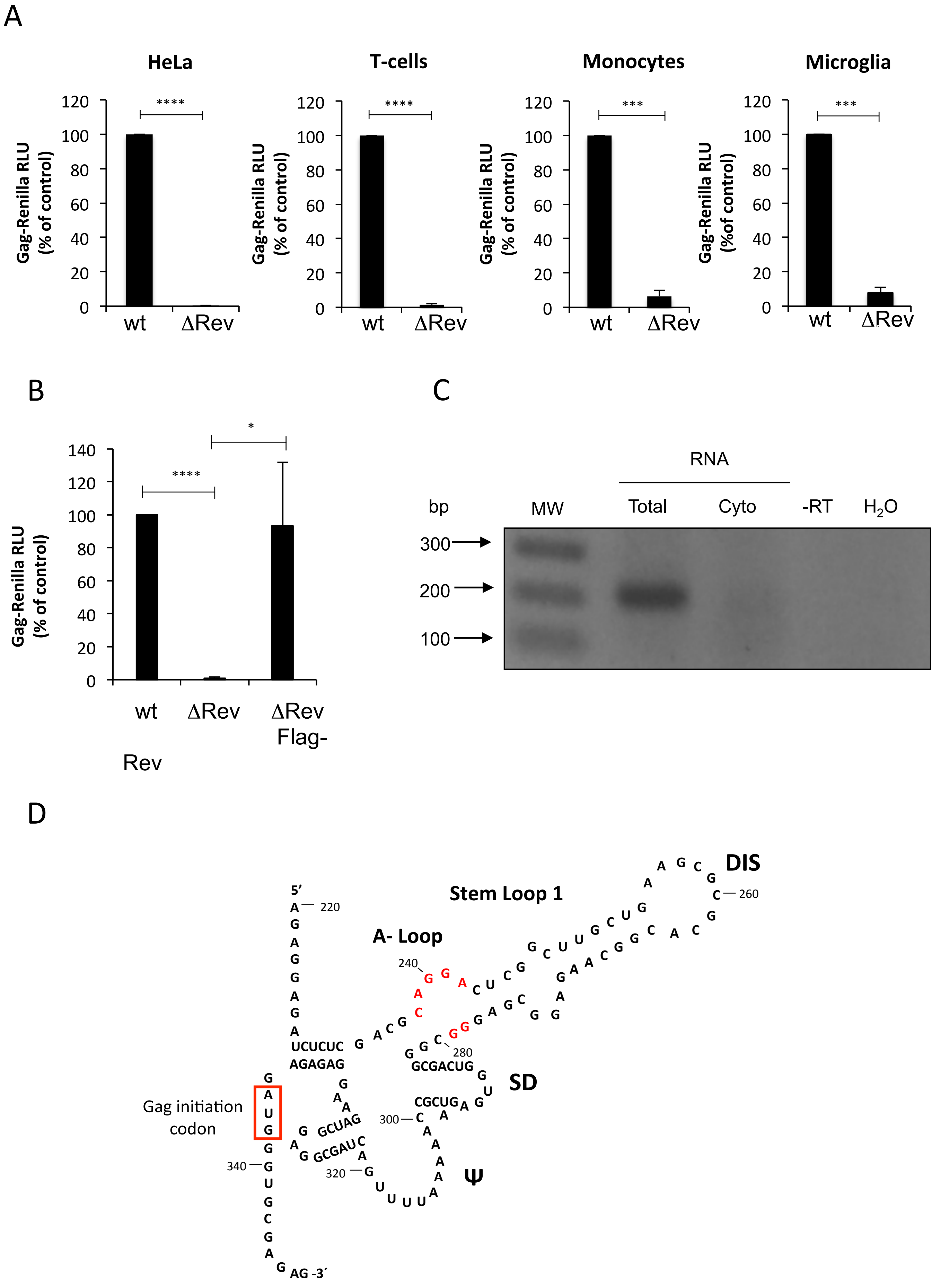

Supplementary Figure 1

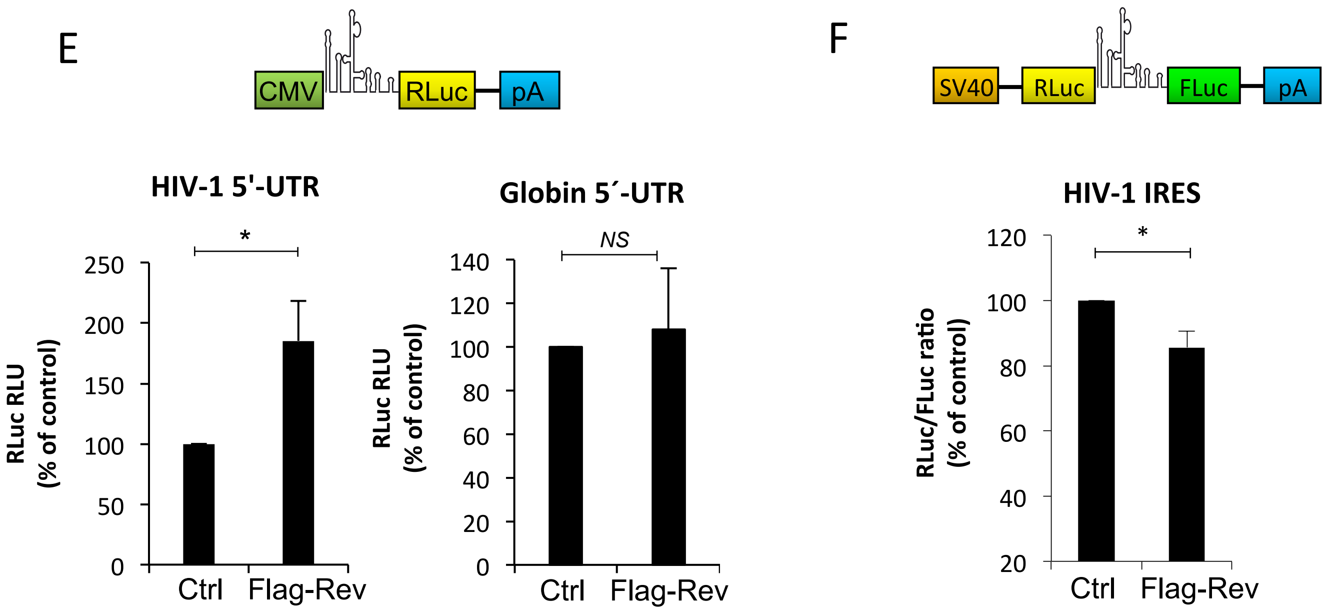

Supplementary Fig. 1: (A) HeLa cells, T-cells (H9 cells) and monocytes (THP-1 cells) were transfected with the pNL4.3R-wt (wt) and pNL4.3R-ΔRev (ΔRev) proviruses as described in materials and methods. Gag-Renilla activity was measured at 24 hours post transfection (hpt). Results were normalized to the wild type provirus (arbitrary set to 100%) and presented as the mean +/- SD of three independent experiments. (***p<0.001; ****p<0.0001, t-test). (B) HeLa cells were transfected with 0.3 µg of pNL4.3R-wt (wt), pNL4.3R-ΔRev (ΔRev) or pNL4.3R-ΔRev together with 0.1 µg of the pCDNA-Flag-Rev vector as described in materials and methods (pCDNA-d2EGFP was used as a control when Flag-Rev was not included). Gag-Renilla activity was measured at 24 hours post transfection (hpt). Results were normalized to the wild type provirus (arbitrary set to 100%) and presented as the mean +/- SD of three independent experiments. (*p<0.05; ****p<0.0001, t-test). (C) pre-GADPH mRNA was amplified by RT-PCR from total and cytoplasmic RNA extracts. Reactions omitting reverse transcriptase (-RT) or Taq DNA polymerase (H_2O) were used as controls. (D) Schematic representation the 5' UTR of HIV-1 showing the A-Loop in the stem loop 1 in the dimerization initiation site (DIS) structure. The splice donor (SD) and Psi (ψ) structures are indicated. The Gag initiation codon is boxed. (E) HeLa cells were transfected with 0.3 µg of pCDNA-HIV-1 5´-UTR or pCDNA-b-globin-5´-UTR together with 0.1 µg of of the pCDNA-Flag-Rev vector as described in materials and methods (pCDNA-d2EGFP was used as a control). Renilla activity was measured at 24 hours post transfection (hpt). Results were normalized to the control (arbitrary set to 100%) and presented as the mean +/- SD of three independent experiments (*p<0.05; and *NS*, non-significant, t-test). (F) HeLa cells were transfected with the pDualLuc 1-336 vector together with 0.1 µg of the pCDNA-Flag-Rev vector as described in materials and methods (pCDNA-d2EGFP was used as a control). Renilla and Firefly activities were measured at 24 hours post transfection (hpt). Results are presented as the Firefly/Renilla ratio normalized to the control (arbitrary set to 100%) and presented as the mean +/- SD of three independent experiments (*p<0.05, t-test).

Supplementary Figure 1

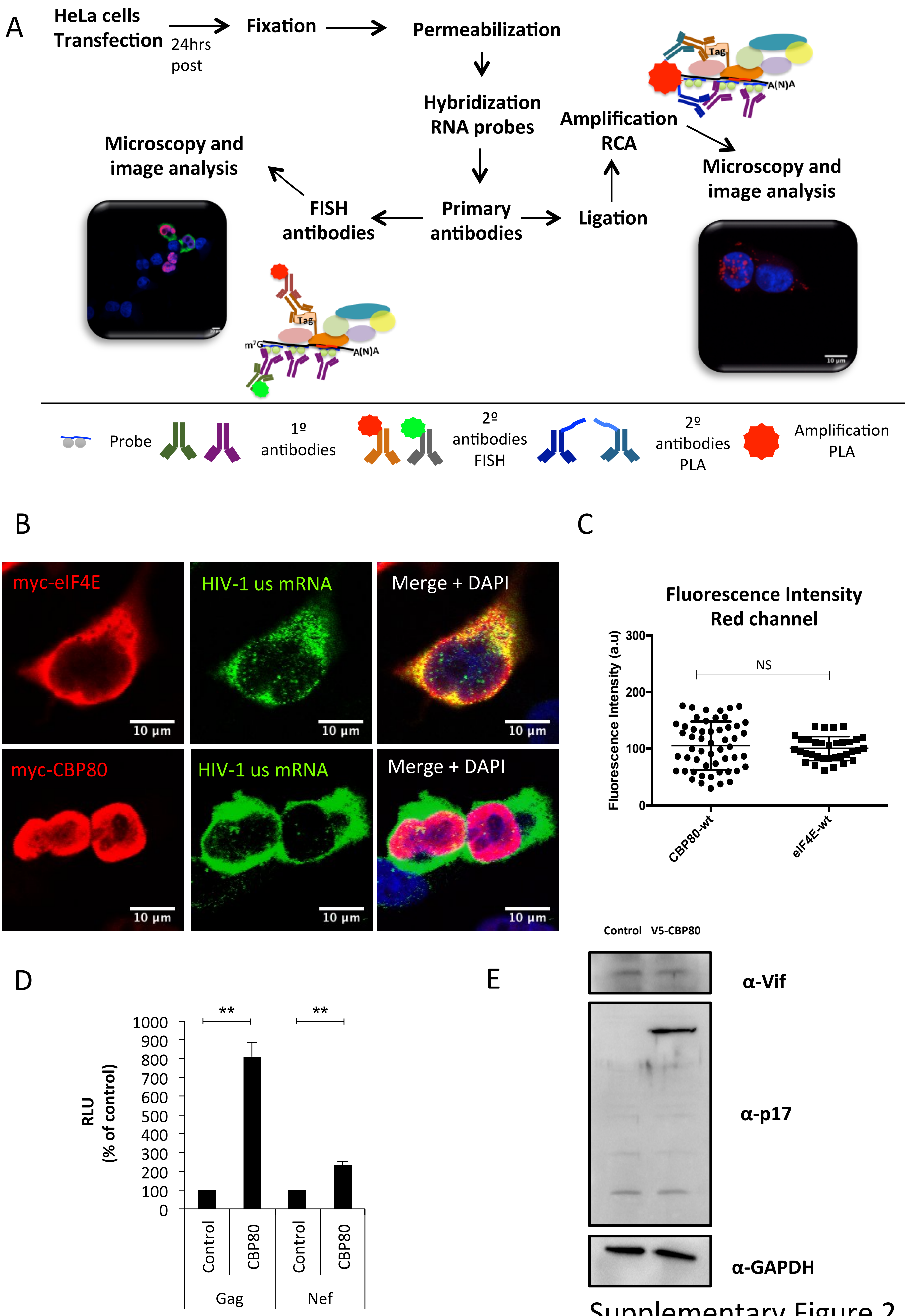

Supplementary Figure 2

F

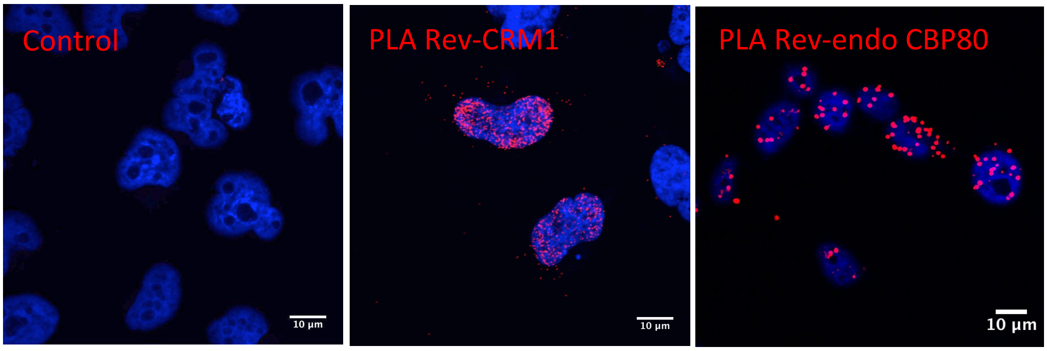

Supplementary Fig. 2: (A) Scheme of the *in situ* hybridization coupled to PLA protocol (ISH-PLA, described in materials and methods). (B) RNA FISH and laser scan confocal microscopy analyzes performed in parallel to the ISH-PLA shown in Fig. 2A. myc-tagged eIF4E and CBP80 are marked in red and the unspliced mRNA is marked in green. Scale bar 10 µm. (C) The intensity of fluorescence of the red channels from myc-tagged protein expression was quantified and plotted (*NS*; non-significant, Mann-Whitney test). (D) HeLa cells were transfected with 0.3 µg of the pNL4.3-RF provirus and with 1 µg of the pCDNA-V5-CBP80 (pCDNA-d2EGFP was used as a control). Gag-Renilla/Nef-Firefly activity was measured at 24 hours post transfection (hpt). Results were normalized to the wild type provirus (arbitrary set to 100%) and presented as the mean +/- SD of three independent experiments. (**$p<0.01$, t-test). (E) HeLa cells were transfected with 0.3 µg of the pNL4.3-RF provirus and with 1 µg of the pCDNA-V5-CBP80 (pCDNA-d2EGFP was used as a control). At 24 hpt, cell extracts were used to detect Gag and Vif by Western blot. Actin was used as a loading control.
(F) HeLa cells were transfected with 1 µg of pCDNA-Flag-Rev or with 1 µg of pCDNA-Flag-Rev and 1 µg pCIneo-HA-CRM1 (untransfected cells were used as a control). At 24 hpt, the interaction between Flag-Rev and endogenous CBP80 or Flag-Rev and HA-CRM1 was analyzed by PLA. Red dots indicate protein-protein interactions. Scale bar 10 µm.

A

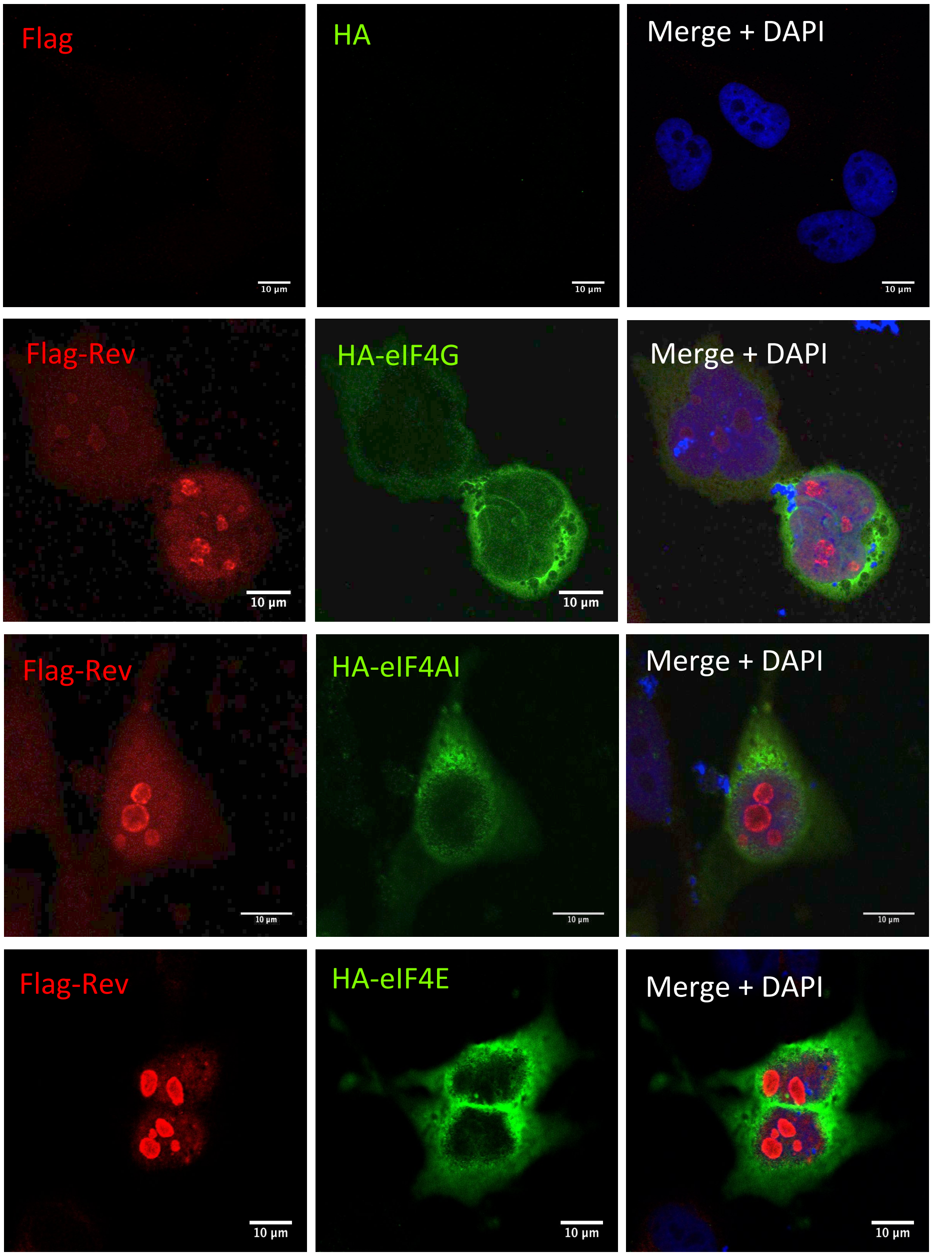

Supplementary Figure 3

B

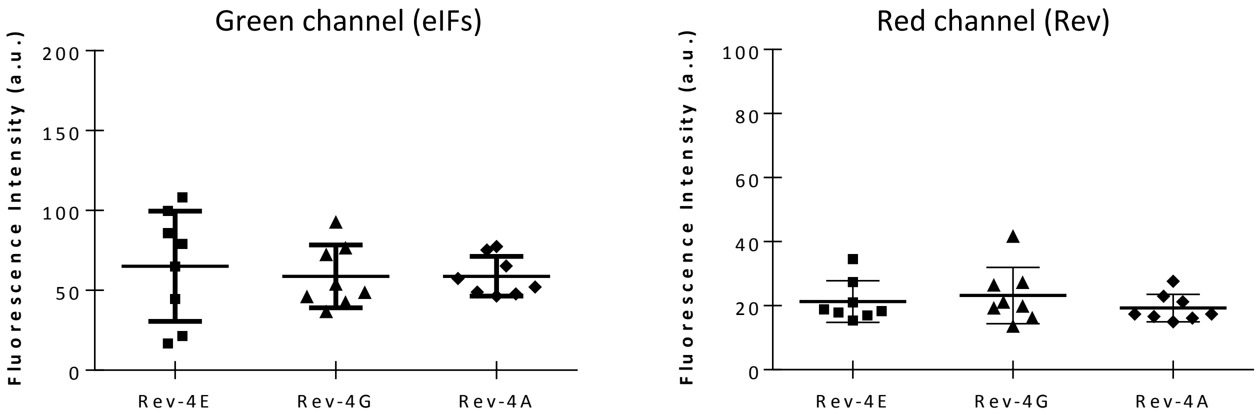

C

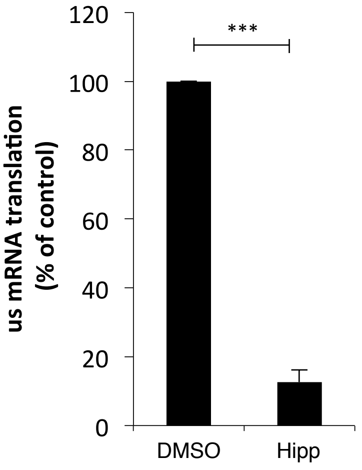

Supplementary Fig. 3: Subcellular localization of Rev and eIF4F components. (A) HeLa cells were either non transfected (upper panels) or transfected with 1 μg of Flag-Rev and 1 μg of HA-tagged, eIF4G, eIF4AI or eIF4E. At 24 hpt, cells were fixed, permeabilized and stained for Flag (red) and HA (green). Images were obtained by confocal microscopy as described in materials and methods. Scale bar 10 mm. (B) The intensity of fluorescence of the green and red channels from representative cells was quantified and plotted. (C) HeLa cells were transfected with 0.3 μg of pNL4.3R and 1 μg of pCIneo-HA-d2EGFP and treated with DMSO(used as a control) or 20 nM hippuristanol overnight. Gag-Renilla activity was measured at 24 hours post transfection (hpt). Results were normalized to the control (arbitrary set to 100%) and presented as the mean +/- SD of three independent experiments. (****p*<0.001, t-test).

A

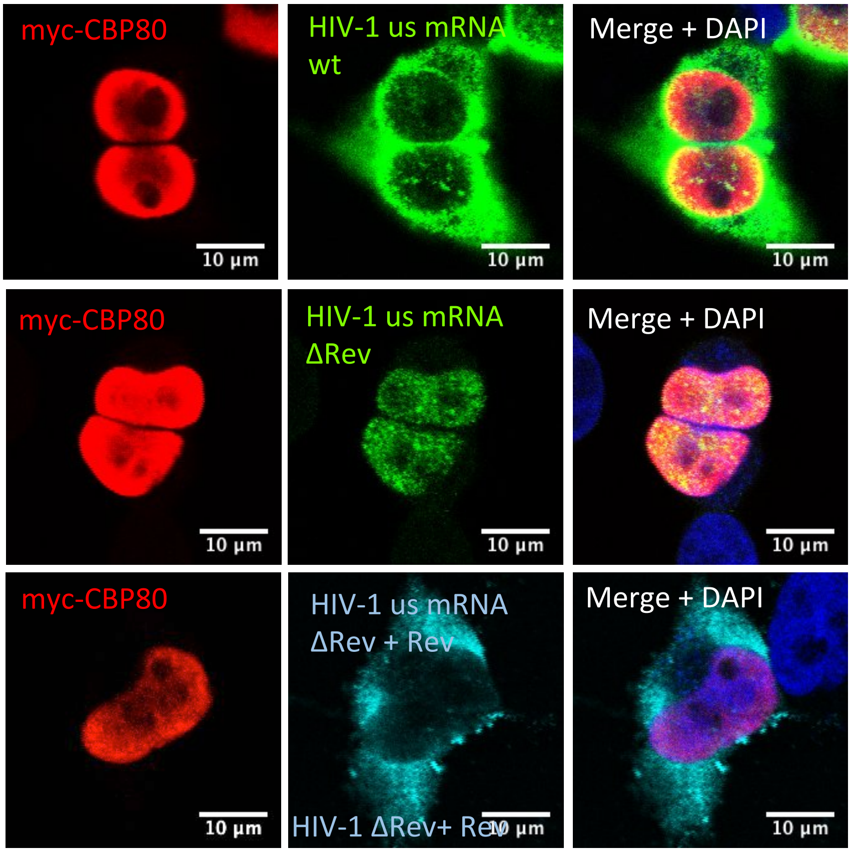

B

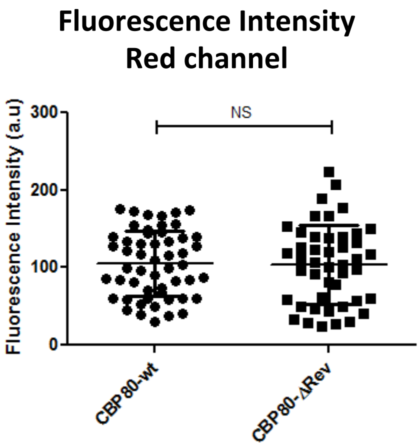

Supplementary Figure 4

C

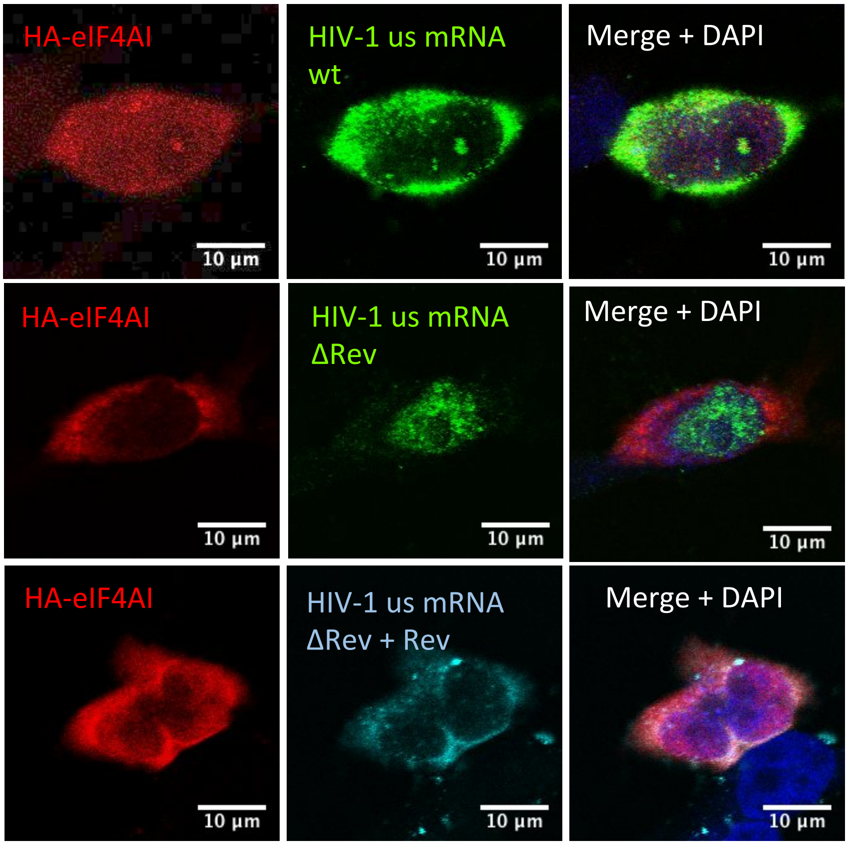

D

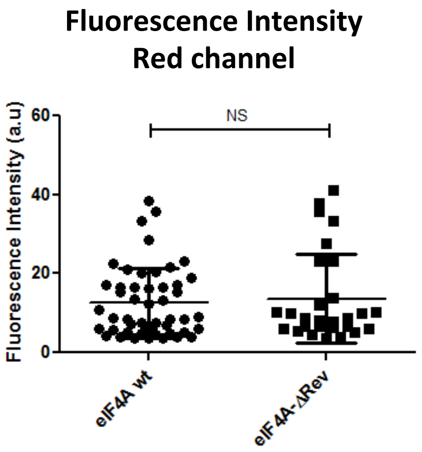

E

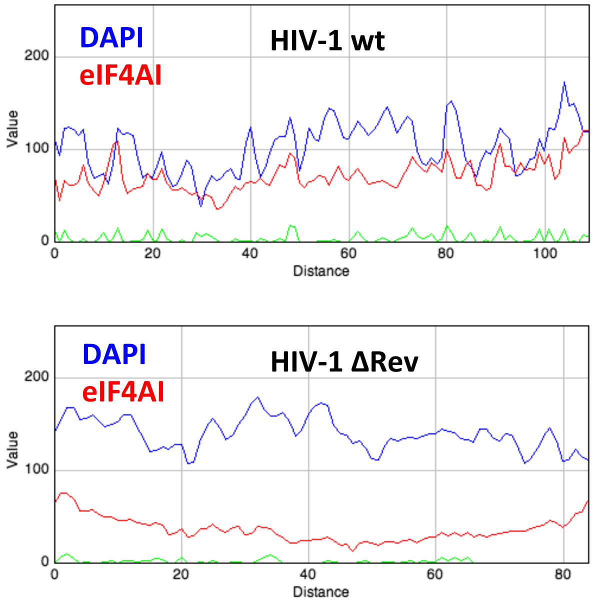

Supplementary Figure 4

Supplementary Fig. 4. (A) RNA FISH and laser scan confocal microscopy analyzes performed in parallel to the ISH-PLA shown in Fig. 4A. myc-tagged CBP80 is marked in red and the unspliced mRNA is marked in green. Scale bar 10 μm. (B) The intensity of fluorescence of the red channel from myc-tagged protein expression was quantified and plotted (*NS*; non-significant, Mann-Whitney test). (C) RNA FISH and laser scan confocal microscopy analyzes performed in parallel to the ISH-PLA shown in Fig. 4B. HA-tagged eIF4AI is marked in red and the unspliced mRNA is marked in green. Scale bar 10 μm. (D) The intensity of fluorescence of the red channels from HA-tagged protein expression was quantified and plotted (*NS*; non-significant, Mann-Whitney test). (E) Intensity plots obtained from the nuclear signals of eIF4AI (red lines) and DAPI (blue lines) in the presence of the HIV-1 wild type (upper panel) and HIV-1 ΔRev (lower panel).

Supplementary Figure 4

A

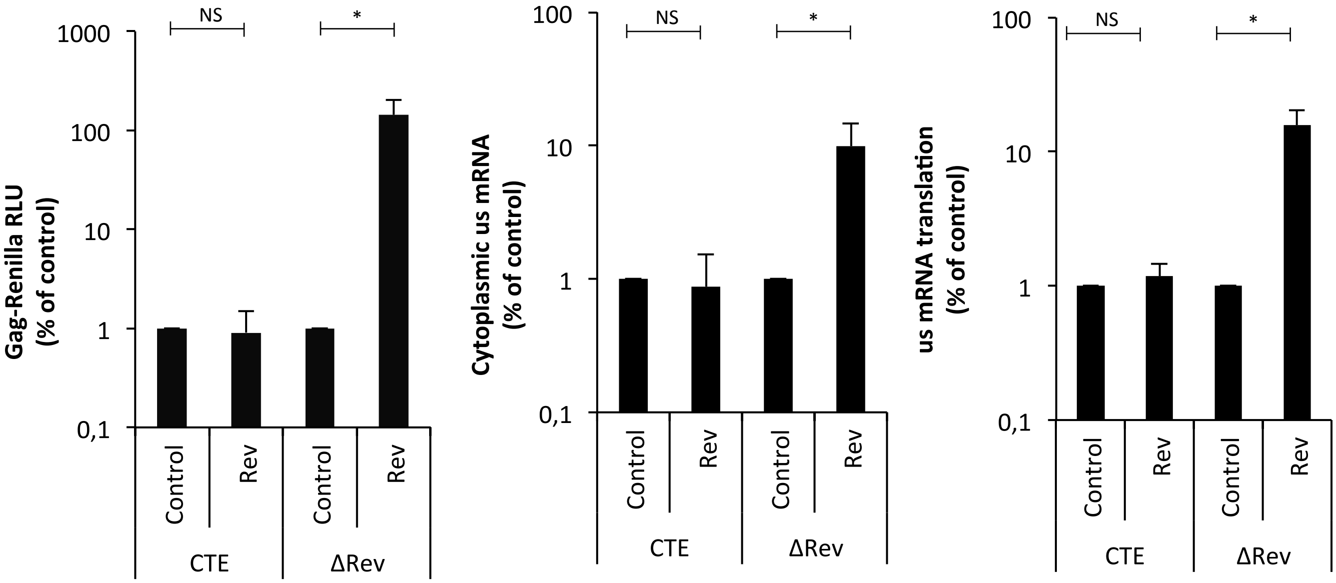

B

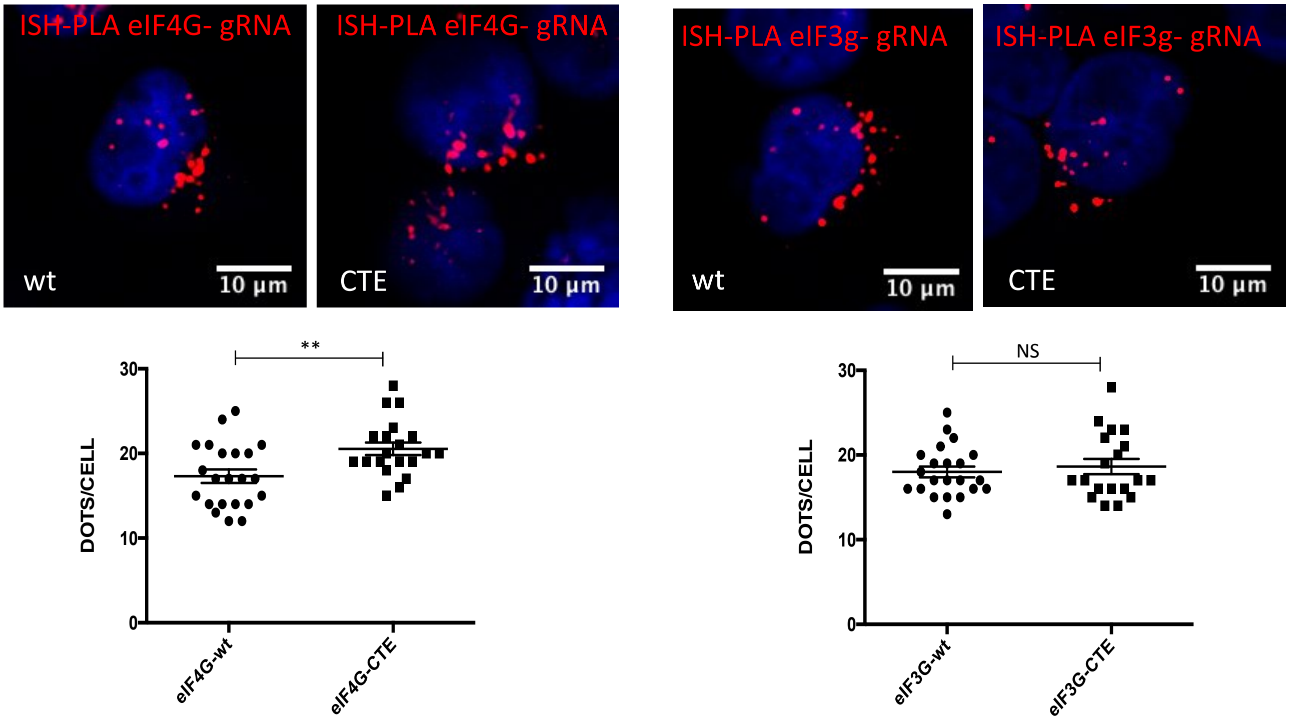

Supplementary Figure 5

Supplementary Fig. 5: (A) HeLa cells were transfected with pNL4.3R-ΔRev or pNL4.3R-CTE together with 0.1 µg of the pCDNA-Flag-Rev vector (pCDNA-d2EGFP was used as a control). At 24 hpt, cell extracts were prepared for Gag-Renilla activity measurement and for cytoplasmic RNA extraction and RT-qPCR analyses. Results for Gag synthesis, cytoplasmic unspliced mRNA and translational efficiency were normalized to the control (arbitrary set to 100%) and presented as the mean +/- SD of three independent experiments. (*$p<0.05$; and NS, non-significant, t-test) . (B) HeLa cells were transfected with 1 µg pNL4.3-wt or 1 µg pNL4.3-CTE together with 1 µg of HA-tagged, eIF4G or eIF3g. At 24 hpt, the interaction between unspliced mRNA and eIF4G or eIF3g were analyzed by ISH-PLA. Scale bar 10 µm (upper panel). Dots per cell quantifications for total unspliced mRNA- eIF4G or eIF3G interactions (lower panel). All interactions were quantified using ImageJ (****$p<0.0001$ and *NS*; non-significant, Mann-Whitney test).

Supplementary Figure 5
